# GPR120/FFAR4 Pharmacology: Focus on Agonists in Type 2 Diabetes Mellitus Drug Discovery

**DOI:** 10.1021/acs.jmedchem.0c01002

**Published:** 2021-04-10

**Authors:** Gabriele Carullo, Sarah Mazzotta, Margarita Vega-Holm, Fernando Iglesias-Guerra, José Manuel Vega-Pérez, Francesca Aiello, Antonella Brizzi

**Affiliations:** † Department of Biotechnology, Chemistry, and Pharmacy, DoE 2018-2022, University of Siena, Via Aldo Moro 2, 53100 Siena, Italy; ‡ Department of Pharmaceutical Sciences, University of Milan, Via Luigi Mangiagalli 25, 20133 Milano, Italy; § Department of Organic and Medicinal Chemistry, Faculty of Pharmacy, University of Seville, Profesor García González 2, 41012 Seville, Spain; ∥ Department of Pharmacy, Health and Nutritional Sciences, DoE 2018-2022, University of Calabria, Edificio Polifunzionale, 87036 Rende, Cosenza, Italy

## Abstract

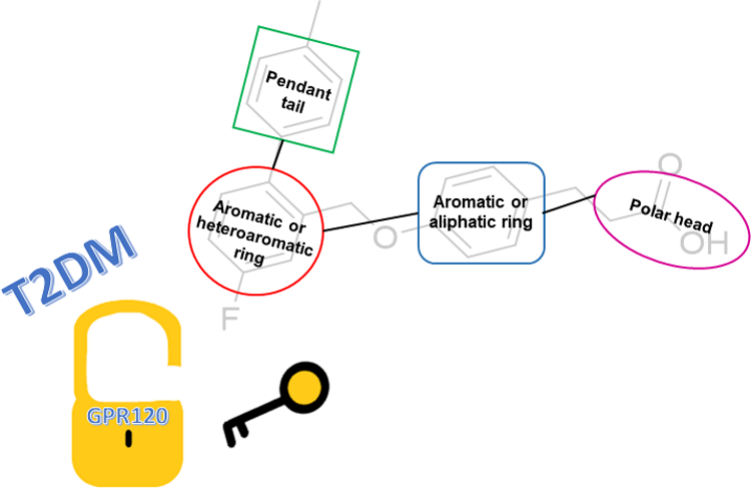

The G-protein coupled receptors (GPCRs) activated by free fatty acids (FFAs) have emerged as new and exciting drug targets, due to their plausible translation from pharmacology to medicines. This perspective aims to report recent research about GPR120/FFAR4 and its involvement in several diseases, including cancer, inflammatory conditions, and central nervous system disorders. The focus is to highlight the importance of GPR120 in Type 2 diabetes mellitus (T2DM). GPR120 agonists, useful in T2DM drug discovery, have been widely explored from a structure–activity relationship point of view. Since the identification of the first reported synthetic agonist TUG-891, the research has paved the way for the development of TUG-based molecules as well as new and different chemical entities. These molecules might represent the starting point for the future discovery of GPR120 agonists as antidiabetic drugs.

## 1 Introduction

Type 2 diabetes mellitus (T2DM) is one of the metabolic diseases that is expected to see a doubling in global prevalence to over 350 million people worldwide in the first three decades of the third millennium.^1^ The typical drugs used to treat T2DM include biguanides, sulfonylureas, thiazolidinedione-derived drugs, and dipeptidyl-peptidase IV (DPP-IV) inhibitors. Over the years, new drugs have emerged such as sodium-glucose cotransporter-2 (SGLT2) inhibitors, which decrease the reabsorption of glucose in the kidney and, therefore, lower blood sugar,^2^ and glucagon-like peptide-1 (GLP-1) receptor agonists, such as exenatide, liraglutide, and lixisenatide.^3^ GLP-1 receptor agonists can promote the functional role of GLP-1, a gut hormone produced by the small intestine in response to oral ingestion of glucose, which promotes a glucoregulatory effect by increasing insulin and suppressing glucagon secretion.^4^ Furthermore, DPP-IV inhibitors showed an interesting therapeutic behavior reducing glucagon levels consisting of the ability to promote incretin secretion in turns. Specifically, these drugs were able to reduce blood glucose fluctuations with an enhancement of GLP-1 preservation and expansion of β-cell mass through the inhibition of apoptotic pathways. These effects are related to better blood glucose control without inducing hypoglycemia.^5^ Since ancient times, the use of appropriate foods as medicines to treat T2DM have emerged both as indigenous remedies or ethnopharmacological tools.^6^ In particular, dietary oils have appeared as interesting ingredients in treating metabolic disorders, as revealed by epigenetic studies.^7^ Specifically, extra virgin olive oil is now considered a useful tool in T2DM treatment, being a GLP-1 secretagogue.^8^ The effects of dietary oils are due to the presence of free fatty acids (FFAs) or their ester forms (triglycerides), which serve as essential nutrients,^9^ and they also act as vital molecules in various cellular processes.^10^ FFAs consist of a carboxylic head connected to a variable aliphatic chain length, which is the typical feature of the different classes: short chain fatty acids (SCFAs) are those with 6 or fewer carbon chains, medium chain fatty acids (MCFAs) have 7–12 carbon chains, and long chain fatty acids (LCFAs) are characterized by longer carbon chains. Furthermore, FFAs vary in the number of unsaturation, generally classified into the saturated, monounsaturated, and polyunsaturated (PUFA) ones.^11^

FFAs have been identified as suitable G-protein coupled receptor (GPCRs) ligands.^12^ These receptors represent the largest human protein family, constituted by seven transmembrane helical domains (TMDs 1–7) linked through three extracellular and three intracellular loops, named helices.^13^ Over the years, GPCRs activated by FFAs have appeared as new and exciting drug targets, due to their plausible shift from pharmacology to therapeutic benefit.^14^ The members discovered were GPR40, GPR41, GPR43, and GPR120, later named FFAR1, FFAR3, FFAR2, and FFAR4 respectively.^15^ GPR40 and GPR120 are activated by MCFAs and LCFAs, while GPR43 and GPR41 are activated by the SCFAs.^12^ The GPR40, overexpressed in pancreatic β-cells,^16^ was the first FFAR to be deorphanized in 2003 and identified as an insulin secretion promoter. Several ligands of this receptor have been identified as potential antidiabetic agents, endowed also with wound-healing properties.^17−20^ In contrast, knowledge about GPR41 and GPR43 is still limited.^21^ Some studies reported their expression in microglia or neurons (especially GPR41), but more information can be detected in various cancer chemotypes^22^ or inflammatory conditions.^23^ GPR120 has been widely studied since its discovery^24^ and deorphanization, demonstrating how FFAs were able to promote incretin secretion by targeting this receptor.^25^ In this perspective, the latest findings about GPR120 and its involvement in several diseases, including cancer, inflammatory conditions, neuroprotection, and, especially, T2DM, are described. From a medicinal chemist point of view, a particular emphasis was given to GPR120 agonists’ usefulness in T2DM management, with a discussion on the structure–activity relationships (SARs) that also includes the patent literature.

## 2 GPR120: Structure, Pharmacology, and Distribution

**Figure 1:**
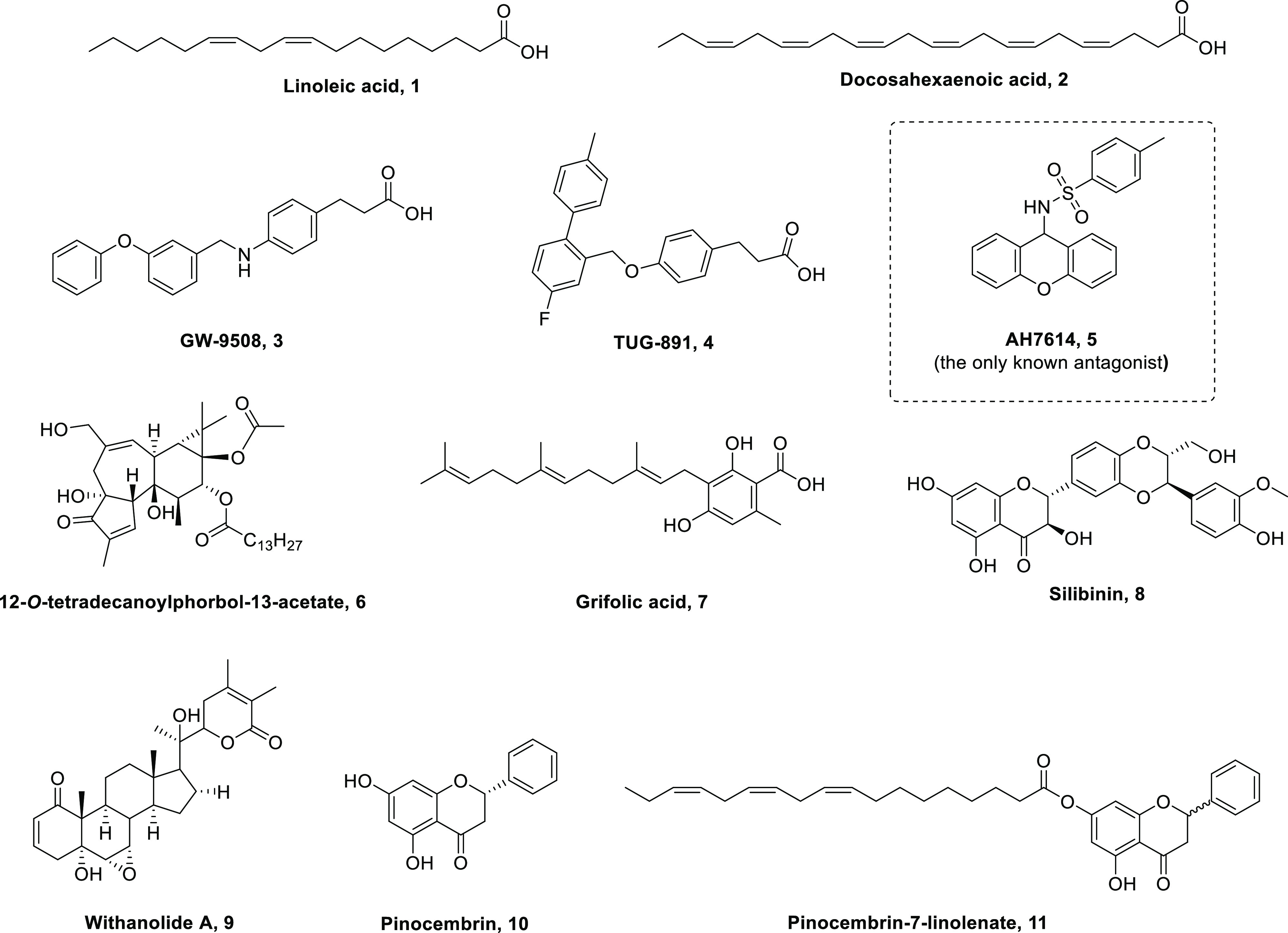
Known GPR120 ligands: part I.

Human GPR120 is a typical GPCR, with a 10q23.33 chromosomal location, constituted by the typical TMDs 1–7, in which the residue Arg99 at the top of TMD2 and Arg178 at the top of TMD4 are the active sites, producing essential interactions for agonist activity.^26,27^ The endogenous GPR120 ligands proved to be PUFAs, including linoleic acid **1** and docosahexaenoic acid **2**, (DHA, Figure 1).^28^ Intriguingly, human GPR120 exists in two splice variants: a short isoform known as GPR120S (Q5NUL3-2, contains 361 residues) and a long isoform known as GPR120L (Q5NUL3, contains 377 residues). The main difference between the two splice variants is the presence of 16 amino acids, between 231 and 247, in the third intracellular loop ICL3 of GPR120L, responsible for different signaling properties (Figure 3).^29^

**Figure 2:**
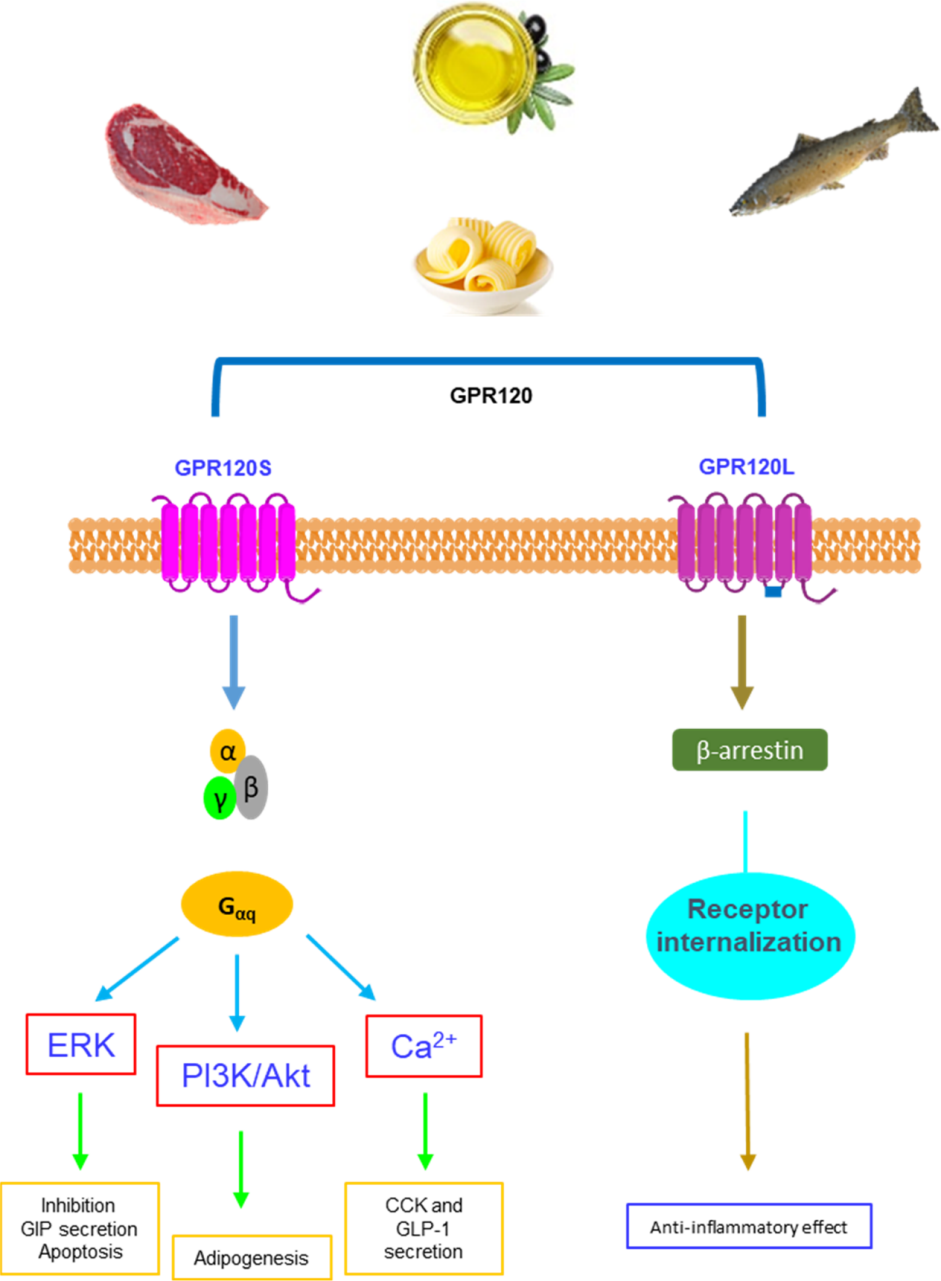
GPR120 pharmacology.

GPR120S is coupled to G_q_/G_11_ as well as to the β-arrestin pathway, promoting Ca^2+^ mobilization, while GPR120L lost its ability of coupling to G_q_/G_11_ but retained its capacity of activating the β-arrestin pathway.^30^ GPR120S transduction is instead limited by inositol triphosphate inhibitors, suggesting the coupling with Ca^2+^ signaling via G_αq_/_11_. Similarly to GPR120S, GPR120L was able to recruit β-arrestin proteins, with a subsequent robust internalization and degradation.^31^ Although phosphorylation of extracellular-signal-regulated kinase 1/2 (ERK1/2) is a typical feature of G-proteins and arrestins, rapid ERK1/2 phosphorylation via G_αq_/_11_ signaling, followed by transactivation of epidermal growth factor receptor, was observed for both GPR120 isoforms.^32^ A strong internalization was observed upon agonist binding through interaction with arrestins. This phosphorylation status involves a series of serine and threonine residues within the intracellular C-terminal tail of the receptor designated cluster 1 (Thr347, Thr349, and Ser350) and cluster 2 (Ser357 and Ser36). The recruitment of β-arrestin 2, the receptor internalization, and the activation of protein kinase B (PKB or Akt) were regulated by GPR120 phosphorylation.^33^ This feature, accompanied by genetic polymorphisms, is responsible for several inflammatory conditions and also for obesity and insulin resistance.^34^ Interestingly, the expression of GPR120 mRNA in delta cells isolated from both healthy individuals and those with T2DM was high.^34,35^ The role of arrestins in GPR120 pharmacology remains to be fully elucidated because of the complex regulation of the ERK1/2-mitogen-activated protein kinases (MAPKs) pathway (Figure 2). Nevertheless, it was not identified a substantial role of the ERK1/2 pathway in HEK293 cells (known for having a lack of expression of either G_αq_ plus G_α11_). Moreover, the key role of the GPR120–arrestins couple was to desensitize this specific pathway, which resulted in the generation of repetitive “spikes” of Ca^2+^ with maintained exposure to an agonist.^36^ GPR120 is abundantly expressed in entero-endocrine cells, including L and I cells. In addition, GPR120 is expressed in macrophages, adipocytes, taste buds, and the gastrointestinal tract, but not found in pancreatic β-cells (it is expressed in α- and δ-cells) (Figure 2).^37,38^ GPR120 is also highly expressed in taste bud cells of circumvallate, fungiform, and foliate papillae. GPR120 colocalizes with the cluster of differentiation CD36, while a limited expression was observed in resident macrophages (Kupffer cells) in the liver.^39^ Different factors, species, and/or strain-dependent factors influenced the expression of GPR120 in the intestine; notably, it is upregulated in the diet-induced obesity rat model.^21^

## 3 GPR120 Physiological Functions and Pharmacological Implications

The high expression of GPR120 in the gastrointestinal tract has drawn attention to itself as a suitable target aimed at investigating new pharmaceutical agents useful in metabolic disorders. Some of its outstanding pharmacology includes interesting secretagogue hormones and having anti-inflammatory, neuroprotective, antiproliferative, and antidiabetic properties.^12,21^

### 3.1 Neuroprotective Functions

GPR120 expression has been detected in the central nervous system, but there is still much to understand about its physiological functions. GPR120 lies in the microglia and hypothalamus where, in cooperation with GPR40, is able to regulate energy homeostasis and inflammation, when combined with a high-fat diet.^40^ Noteworthy, **2** prevented the inflammatory state in a model of neuroinflammation induced by tumor necrosis factor-α (TNF-α) in the rHypoE-7 cells (Figure 1); nevertheless, this effect was Akt/ERK independent and instead related to the activation of transforming growth factor-β-activated kinase 1 binding protein.^41^ Interestingly, GPR120 is also expressed in gonadotrophs of the mouse’s anterior pituitary gland. The expression of the receptor GPR120 is directly regulated by female hormones in the reproductive cycle at the pituitary level.^42^ In a model of middle cerebral artery occlusion, in which the inflammation was induced by oxygen-glucose deprivation, **2** was able to facilitate GPR120 activation recruiting β-arrestin. **2** also provided protection against focal cerebral ischemic injury through a combination of anti-inflammatory (reduction of IL-1β, IL-6, and TNF-α) and antiapoptotic effects, via a decrease in B-cell lymphoma of the 2/Bcl-2-associated X protein (Bcl-2/Bax).^43^ Remarkably, in a model of laser-induced choroidal neovascularization (CNV), GPR120 activation was able to suppress CNV, while also reducing inflammation markers (IL-6, IL-1β) via the nuclear factor kappa B (NF-κB) pathway in the retina.^44^ In a typical neuronal dysfunction, such as subarachnoid hemorrhage (SAH)-induced early brain injury, 2 weeks before oral gavage at 1 g/kg body treatment of fish oil (30% PUFAs) suppressed SAH-induced brain cell apoptosis and neuronal degradation. GPR120 activation also rescued behavioral impairment and brain edema, through the regulation of the GPR120/β-arrestin 2/TAK1 binding protein-1 pathway.^45^

### 3.2 Antiproliferative Functions

The presence of LCFAs in foods highlighted their role in human nutrition, including anti-inflammatory and cancer-preventive activities, with a particular emphasis on the gastrointestinal tract.^46−50^ Scientific evidence demonstrated that fat nutrients are involved in cancer because FFAs targeted GPR120,^51^ showing a relationship with colorectal cancer, but also with melanoma, lung, prostate, and breast cancers.^52^ In breast tissues, GPR40 and GPR120 are both expressed. Their role was investigated in MCF-7 and MDA-MB-231 cell lines. In these cell lines, lysophosphatidic acid (LPA) and epidermal growth factor (EGF) were used to induce ERK/Akt activation, resulting in cell proliferation. LCFAs inhibited this effect; likewise, the synthetic GPR120 agonists **3** and **4** (Figure 1) inhibited LPA- and EGF-induced proliferation, demonstrating the predominant role of GPR40 in this effect.^53^ Moreover, in the MDA-MB-231 cell line, **1** was used to induce proliferation, thanks to serine/threonine kinase 2 phosphorylation. The migration in this case was inhibited by the treatment with the selective GPR120 antagonist AH7614 **5** (Figure 1), although the exact mechanism was not completely investigated.^54^ Further studies demonstrated how GPR120 is an independent prognostic factor for recurrences in hormone receptor-positive breast cancer. In particular, GPR120 activation mediated by endogenous ligands or the synthetic **4** increased tamoxifen resistance, which is dependent on ERK/Akt pathways, whereas GPR120 knockdown or antagonist **5** abrogated this effect.^55^ Although GPR120 is overexpressed in lung tissues, few studies reported its involvement in lung cancer. Specifically, in rat RLCNR, mouse LL/2, and human A549’s lung cancer cells, it was shown how GPR40 agonist activity was able to promote metastasis, while GPR120 negatively regulated lung tumor progression.^56^ The same effects were observed in melanoma cells stimulated by the GPR120 agonist 12-*O*-tetradecanoylphorbol-13-acetate **6** (Figure 1).^57^ In contrast with the results obtained in lung and skin tissues, GPR120 promoted cell motile activity and progression of the osteosarcoma MG63-R7 cell line; on the other hand, GPR40 antagonists suppressed this effect.^58^ In prostate PC-3 and DU145 cell lines, EPA and **2** inhibited the LPA-induced proliferation, interfering with the phosphorylation and subsequent activation of ERK1/2, protein tyrosine kinase 2, and p70S6K. Indeed, in DU145 cells, **2** inhibited LPA-induced proliferation with an IC_50_ of 73 nM, compared to a relative potency of 5.7 μM for grifolic acid **7** (Figure 1).^59^ In this field, GPR120 agonists were, in turn, able to inhibit proliferation by suppressing positive cross-talk between LPA and the EGF receptors.^60^ Other studies showed a central role in the antiprostate cancer effects of dietary LCFAs, mediated through inhibition of M2-like macrophages.^61^ GPR120 is also expressed in Hep3B and HepG2 human hepatoma cells, where its activation by **2** inhibited lipid accumulation induced by the liver X receptor activator T0901317.^62^ Specifically, **2** was able to provide protection from steatosis by activating GPR120 in hepatocytes. The GPR120 signaling cascade sequentially involved G_q/11_ proteins, with suppression of Ca^2+^/calmodulin-dependent protein kinase, 5′ AMP-activated protein kinase (AMPK), and sterol regulatory element-binding protein 1 expression.^62^ The natural GPR120 agonist **7** dose- and time-dependently induced the necrosis of the rat’s anterior pituitary adenoma GH3 cells. In particular, it significantly reduced the mitochondrial membrane potential (MMP) and decreased cellular adenosine triphosphate levels in GH3 cells.^63^ In pancreatic cancer PANC-1 cells, **3** provoked a significant decrease in migration, but in combination with selective GPR40 antagonist GW1100, this effect was reverted.^64^ Colorectal cancer proved to be the most intriguing for the investigation of GPR120 ligands.^65^ There was a strong clinical-pathological correlation linked to GPR120 expression in human colorectal tissues; interestingly, the expression of the receptor was noted to increase at the clinical stage of cancer, rendering it a suitable tool for the development of anticancer compounds. **3** enhanced mRNA and protein expression of proangiogenic factors including VEGF, IL-8, and COX-2, and this effect was related to GPR120-induced activation of PI3K/Akt-NF-κB signaling, highlighting the possibility to develop GPR120 antagonists as anticancer tools.^66^ In contrast with this study, LCFAs, by activating GPR120, suppressed cell proliferation and promoted apoptosis in colorectal cancer cells.^67^ To identify new potential antiproliferative agents targeting GPR120, a homology model was developed to explore natural products as suitable ligands. From these docking simulations, silibinin **8** and withanolide **9** (Figure 1) have shown good interactions with active site residues of the receptor.^68^ Nevertheless, these data were not confirmed through functional studies, limiting their future development as anticancer agents targeting GPR120.

### 3.3 Wound-Healing Functions

As reported, GPCRs are also abundantly expressed in skin. In this tissue, some members, such as GPR4, GPR65, GPR68, and GPR132, are involved in the wound-healing process.^69^ Also GPR40 activation by quercetin-3-oleate showed interesting wound-healing properties in HaCaT cell line.^18^ In the field of natural products active as wound-healing enhancers, **2** proved to promote wound healing targeting GPR120.^70^ Interestingly, the flavanone pinocembrin **10** (Figure 1) had been demonstrated to promote wound healing too,^20^ but very recently this action has been associated with GPR120. In fact, aiming to ameliorate this aspect, some esters of **10** with fatty acids were synthesized, and among these, its linolenoyl derivative **11** (Figure 1) showed an interesting healing activity potentially involving GPR120 activation. Noteworthily, docking simulation experiments showed that **10** and **11** share the same binding site of **4**.^71^

## 4 T2DM: Role of GPR120 in a Complex Metabolic Disorder

FFARs emerged as important therapeutic targets because fats contained in foods furnish fatty acids as dietary metabolites able to activate FFARs, promoting different physiological/pharmacological functions, especially metabolic ones.^21^ The different members of the family, GPR40-GPR41-GPR43-GPR120, show a complex pharmacology and different functions in the human body, although GPR40 and GPR120 remain the most studied members of the family.^12^

### 4.1 GPR40-GPR41-GPR43-GPR120s Metabolic Functions

**Figure 3:**
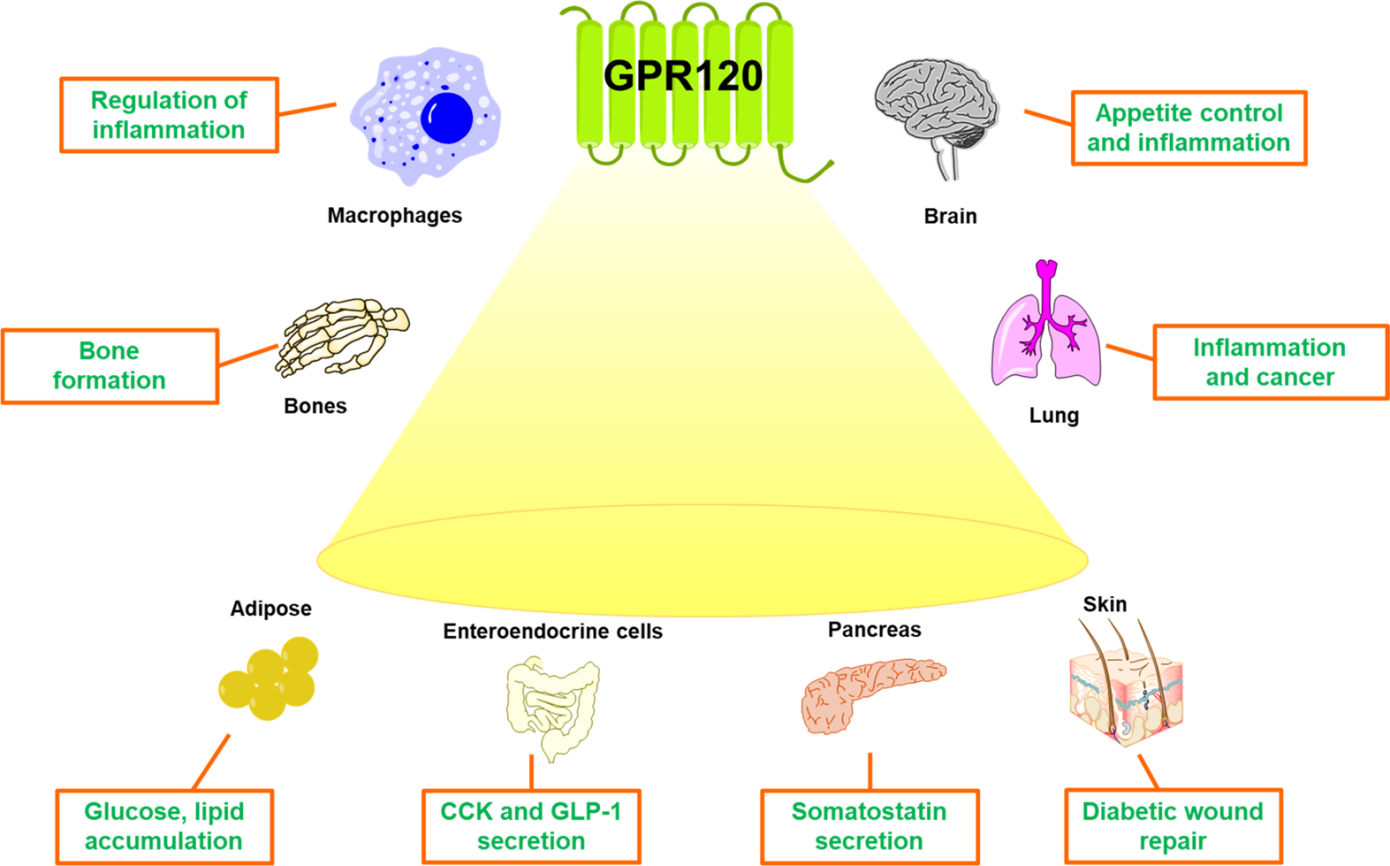
GPR120 spatial distribution and physiological functions in the human body.

GPR41 and GPR43 are generally activated by SCFAs and are related to several metabolic functions.^12^ GPR41 and GPR43 are involved in GLP-1 secretion; GPR43 activation has been shown to improve glucose-stimulated insulin secretion (GSIS), while GPR41 activation is limited in GSIS when it comes to pancreatic β-cells. Nevertheless, few studies have involved them in pharmacological and clinical research around T2DM.^21^ The most studied members, GPR40 and GPR120, only show 10% of homology between their amino acid sequences, but the activation of the two receptors by FFAs is similar.^72^ GPR40 has been shown to promote insulin secretion from the pancreas and GLP-1 secretion from enteroendocrine cells. Clinical studies showed that fasiglifam, known as TAK-875, an agonist of the GPR40 receptor, improved glycemic control and reduced glycated hemoglobin (HbA1c) levels in T2DM patients, reducing the risk of hypoglycemia. However, this ligand was removed from clinical trials due to potential liver toxicity.^73^ Despite its spatial distribution (Figure 3), GPR120 showed involvement in several functions, including secretion of GLP-1 from enteroendocrine cells, inhibition of ghrelin secretion, stimulation of glucose uptake by adipocytes, promotion of pancreatic β-cell survival, and inhibition of pro-inflammatory cytokines release from macrophages.^74^

### 4.2 GPR120 Deorphanization and Ligand-Binding Interactions

In 2003, Fredriksson and colleagues identified GPR120 as a new rhodopsin-like GPCR.^24^ In 2005, Hirasawa and colleagues deorphanized the receptor validating it as a promoter of incretin secretion^25^ and prompted the search for putative ligands starting from a library of over 1000 compounds. Changes in the amount of the internalized fluorescently labeled receptor were examined in the endocytic compartment, by using HEK293 cells, which stably express GPR120-enhanced green fluorescent protein (EGFP). LCFAs were found to evoke specific internalization of the GPR120–EGFP conjugate.^25^

**Figure 4:**
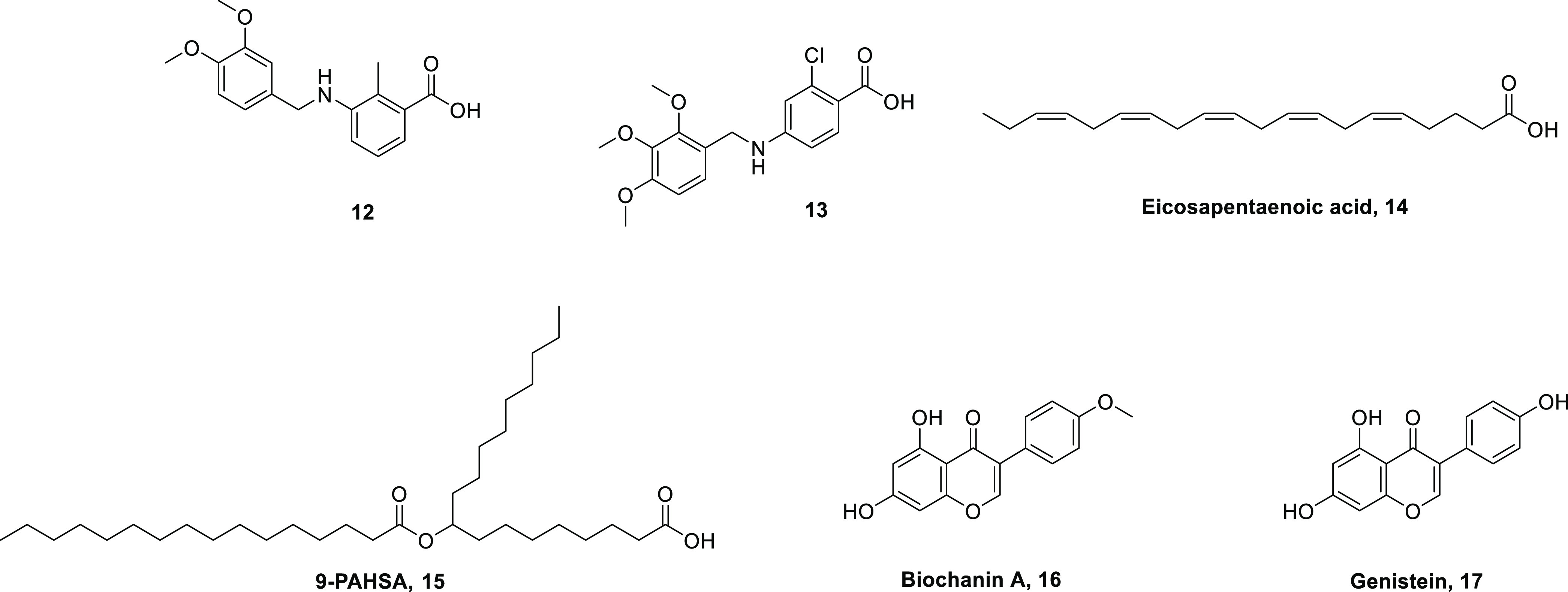
Known GPR120 ligands: part II.

**1** was found to be a potent agonist for this “new” FFA receptor (pEC_50_ = 5.16). Among the endogenous ligands of the receptor, GPR120 ligands were individualized as LCω-3 PUFAs (such as **2**) (Figure 1). However, ω-6 PUFAs are also natural GPR120 ligands. Both ω-3 and ω-6 PUFAs increased the cytosolic concentration of Ca^2+^ and activated the MAPK-ERK1/2 pathway.^28^ After that, further studies were conducted to find new GPR120 ligands. On the basis of **3**, that was demonstrated to be a GPR40/GPR120 dual ligand, the molecule known as **4** (Figure 1) was validated as a powerful selective GPR120 ligand.^75^ To explore the binding mode, the research started from a historical knowledge that arginine (Arg) residues seem to be significantly implicated in FFARs activation. In particular, a single Arg at position 2.64 (amino acid 99 in the primary sequence) was identified as the key residue involved in the interaction between GPR120 and the carboxylate of **1** (Figure 1). Twenty-one residues were then predicted as fundamental for the ligand-binding pocket after its mutagenetic replacement (except for R99Q^2.64^ and F303H^7.35^).^76^ The binding pocket was indicated to be located between TMD2, TMD3, and TMD5–7. Docking studies of ligands demonstrated a strong correlation between the observed potency in a receptor β-arrestin 2 interaction assay and calculated relative binding energies. The mutagenesis study revealed how alanine (Ala) mutations with W104A, F115A^3.29^, F211A^5.42^, W277A^6.48^, and F304A^7.36^ completely abolished the response to **1** and **3** ligands in the receptor β-arrestin 2 interaction assay, validating this model as intriguing because of its accurate description of the GPR120 crystal structure.^27^ The search for suitable hit structures as GPR120 modulators spurred several medicinal chemists to propose new chemical candidates. In this context, Li and colleagues proposed a pharmacophore structure named “Hypo1”.^77^ Hypo1 is constituted by two aromatic rings, one negative ionizable group, and one hydrophobic substituent. Starting from FFAs, 50 different compounds were identified from a virtual library, and the screened compounds were then overlapped into all four pharmacophore’s points. Considering that FFAs can activate the ERK pathway,^78^ this feature was used to analyze their GPR120 agonist activity. Hypo1-derived compounds **12** and **13** (25 and 50 in the reference) (Figure 4) showed the best β-arrestin 2-based property, which was structurally related to a benzylic residue in the molecule (Figure 4).^79^ This pharmacophore model was also validated by the data obtained with **1**’s methyl ester, which docked in the active site of GPR120, but unfortunately, it turned out to be inactive *in vitro*, probably because of the distance between the oxygen of the carboxylate of **1**’s methyl ester and nitrogen of the guanidine in Arg99 (7.01 Å).^80^ It is clear that further biochemical and homology modeling studies need to be conducted to understand properly the receptor structure.

### 4.3 Appetite Control and Gut Hormone Secretion

Appetite and gut hormone secretion are important features in controlling hyperglycemia associated with T2DM. Food intake and energy balance are centrally regulated by neuropeptide Y in the arcuate nucleus, which stimulates food intake and inhibits energy expenditure. GPR120 is highly present in the epithelium of the circumvallate papillae, where it functions as a sensor for dietary fats but also is coexpressed in neurons which manifest neuropeptide Y in the arcuate nucleus. Consequently, GPR120 activation could suppress food intake.^81^

GPR120 activation, mediated by administration of **4**, had an anorectic effect, according to the data obtained with LCFAs; this result demonstrated how GPR120 activation reduced appetite via a partially neuropeptide Y inhibition.^30,82^ Furthermore, GPR120 is involved in the release of ghrelin, a neuropeptide secreted in gastric cells; a consequent increase of ghrelin plasma concentration is observed during fasting. Unsaturated LCFAs (**2**, linolenic acid, and palmitoleic acid) were able to inhibit ghrelin secretion activating GPR120; the same feature was observed for synthetic agonist **3**.^83^ Further investigation showed how this inhibition was mediated by G_i/0_ proteins, although the exact mechanism is yet to be demonstrated, taking also into account the limited role of G_i/0_ proteins in GPR120 signal transduction.^84^ GPR120 is involved in the release of cholecystokinin, a hormone implied in the release of insulin from the pancreas but also of digestive enzymes. Moreover, GPR120 coparticipates with monovalent cation-specific transient receptor potential channel type M5 to improve cholecystokinin release, with a resulting increase in intracellular Ca^2+^ concentration.^85^ Overall, GPR120 is responsible for transmitting taste sensations and modulating taste preferences in response to the presence of fats. The receptor is also implicated in the cross-talk to sweet taste preference via secretion of lingual GLP-1.^86^

### 4.4 Adipogenesis

GPR120 is present in adipose tissue, and its expression is related to an increase during adipocyte differentiation. GPR120 knockout (KO) 3T3-L1 cells led to a reduction of adipocyte markers, validating the previous observation.^87^ LCFAs enhanced glucose uptake via GPR120 in cultured 3T3-L1 adipocytes, with a G_q/11_-dependent mechanism. The ω-3 PUFA eicosapentaenoic acid (EPA, **14**, Figure 4) regulated the expression of vascular endothelial growth factor-A in 3T3-L1 adipocytes through the activation of both GPR120 and peroxisome proliferator-activated receptor-gamma (PPARγ), which may be important for the vascularization of adipose tissue and key to a significant reduction in gene and protein levels of insulin receptor substrate 1 and glucose transporter type 4.^88^ In order to fully explain the possible molecular mechanism, it was shown how the increased PPARγ expression is accompanied by an increase in the [Ca^2+^]i and ERK1/2 signal pathway.^89^ This behavior was also demonstrated in high-fat, diet-fed (HFD) and GPR120-deficient mice, in which a decreased adipocyte differentiation and lipogenesis compared to wild-type animals were observed.^34^ However, in adipocytes GPR120 expression was inhibited by inflammatory markers, limiting the possibility to explore the efficacy of GPR120 agonists in inflammation-induced obesity.^90^ Besides, 9-[(1-oxohexadecyl)oxy]-octadecanoic acid (9-PAHSA, **15**, Figure 4) and endogenous FFAs induced browning of 3T3-L1 adipocytes via enhanced expression of brown fat specific genes. These effects are mediated by GPR120 activation, which in turns inhibits LPS/NF- κB cascade, highlighting the possibility to investigate the role of GPR120 antagonists in treating obesity.^91^

### 4.5 Anti-Inflammatory Functions

Inflammation is usually associated with impaired β-cell function and reduction of insulin sensitivity. FFAs are well-known anti-inflammatory agents,^92^ and several pieces of evidence show how they exert their activity targeting GPR120.^30^ In particular, LCFAs showed an involvement in several conditions.^93^ It was shown how **2** and **3** were able, via Gα_q_ and β-arrestin 2 transduction, to activate cytosolic phospholipase A2 and cyclooxygenase 2 (COX-2), with the consequent prostaglandin E2 release in RAW264.7 macrophages; this mechanism, covered by a NF-κB signaling pathway is in turn responsible for the anti-inflammatory effect.^94^ The exact mechanism by which **2** was able to induce an anti-inflammatory effect was linked to GPR120/C-Raf-MAPK transduction and increased expression of inducible nitric oxide synthase (iNOS).^95^ This feature, in turn, promoted the expression of several cytokines, such as interleukins (ILs) IL-1β, IL-6, IL-10, IL-12, TNF-α, interferon γ, and tumor growth factor TGF-β. The same pathway was also responsible for the anti-inflammatory effect induced by EPA in the same cell line.^96^ Another LCFA, 10-oxo-*trans*-11-octadecenoic acid (KetoC), suppressed the pro-inflammatory cytokines TNF-α, IL-6, and IL-1β via NF-κB p65 in macrophages by binding GPR120.^97^ Interestingly, **2** also showed anti-inflammatory effects in primary human chondrocytes. Additionally, in a skin defect model of osteoarthritis, **2** enhanced wound repair in mice, as shown by the downregulation of the number of CD68^+^ cells.^98^ On the other hand, **2** suppressed the inflammatory cytokines in the liver tissues and prevented fibrosis in the wild-type mice fed with a choline-deficient HFD diet.^99^ In a model of LPS-induced osteoclast formation, **2** fostered bone resorption by activating GPR120 with the consequent reduced production of TNF-α in macrophages. Nevertheless, **2** directly inhibited osteoclast formation.^100^ These health-promoting effects induced by LCFAs, particularly **2**, prompted scientists to investigate new molecules able to activate GPR120 with the aim of obtaining new anti-inflammatory candidates. Biochanin A **16** and genistein **17** (Figure 4), two natural isoflavones, were compared to LCFAs for their affinity versus GPR120 and PPARγ in *in silico* studies, showing how they represent good tools for the design of new suitable dual ligands, useful in inflammatory conditions.^101^

### 4.6 Antidiabetic Functions

In line with other members of the FFARs family, GPR120 is also involved in a well-orchestrated antidiabetic activity.^102^ From a spatiotemporal point of view, GPR120 is highly expressed in enteroendocrine cells, where its activation by agonists was able to promote incretin (GLP-1) secretion. GPR120 is also expressed in K cells, favoring the secretion of gastric inhibitory peptide (GIP). After GPR120 activation by lard oil, GIP secretion increased with a consequent reduction in plasma glucose levels.^21^ Furthermore, **1** was able to promote GLP-1 secretion after a long-term supplementation, thus promoting pancreatic insulin secretion and β-cell proliferation in rats.^103^ GPR120 deficiency impaired metabolic balance, leading to insulin resistance. GPR120 KO mice showed more severe signs of insulin resistance when fed with an HFD. FFAs were able to enhance muscle and hepatic insulin sensitivity, increase glucose infusion rate, promote hepatic lipid metabolism, and decrease hepatic steatosis in wild-type mice but not in GPR120 KO mice, highlighting its role in T2DM management.^104^ In human islets, GPR120 expression is positively associated with insulin secretion and content but negatively with HbA1c percentage. Pancreatic islets from hyperglycemic or diabetic patients have reduced GPR120 expression compared to healthy individuals. However, GPR40 was also found to directly promote insulin secretion from the pancreas, only partially contributing to the FFA-stimulated insulin secretion. In this field, the role of GPR120 was to mediate FFA-stimulated elevation of [Ca^2+^]i in intestinal cells, an important step in triggering insulin secretion. It has been reported that β-arrestin 2 can play important roles in the regulation of insulin-Akt signaling in the liver and pancreatic islets. To date, selective GPR120 activation regulates both islet and enteroendocrine hormone function with agonist combinational therapy.^105^ All these observations are evidence of how GPR120 might serve as a suitable target for the development of T2DM drug candidates.

## 5 GPR120 Agonists in T2DM Drug Discovery

**Figure 5:**
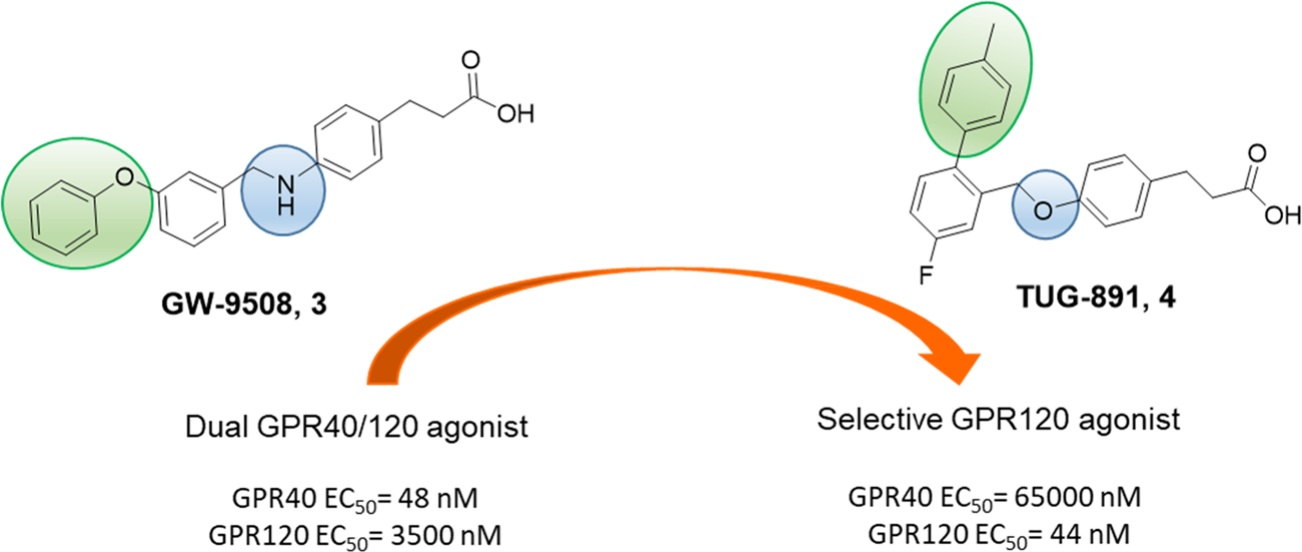
From GPR40 to GPR120 agonists: a structural refinement (in green the shifted position of aromatic portion while in blue the heteroatom change, useful to obtain GPR120 selectivity).

As reported, GPR120 was deorphanized in 2005, and its first ligand **1** (Figures 3 and 5) was demonstrated to favor GLP-1 secretion, highlighting its potential role as an antidiabetic drug target. Over the years, pharmacological studies demonstrated the complex GPR120 pharmacology and, at the same time, promoted the opportunity to target it in metabolic disorders, including T2DM (Figure 2).^106^ The search for synthetic GPR120 agonists started from GPR40 ligands, given the similar amino acidic sequence of the two receptors. Today, it is well-known how a GPR40 agonist also could be a suitable GPR120 agonist.^107^ Starting from the discovery of the selective GPR120 agonist **4** (Figure 5),^75^ various series of new derivatives have been developed as GPR120 agonists, mainly as carboxylic acid derivatives or sulfonamide ones, and assayed as intriguing antidiabetic tools.

### 5.1 Carboxylic-Acid-Derived Agonists

#### 5.1.1 Phenylpropanoic Acids

**Figure 6:**
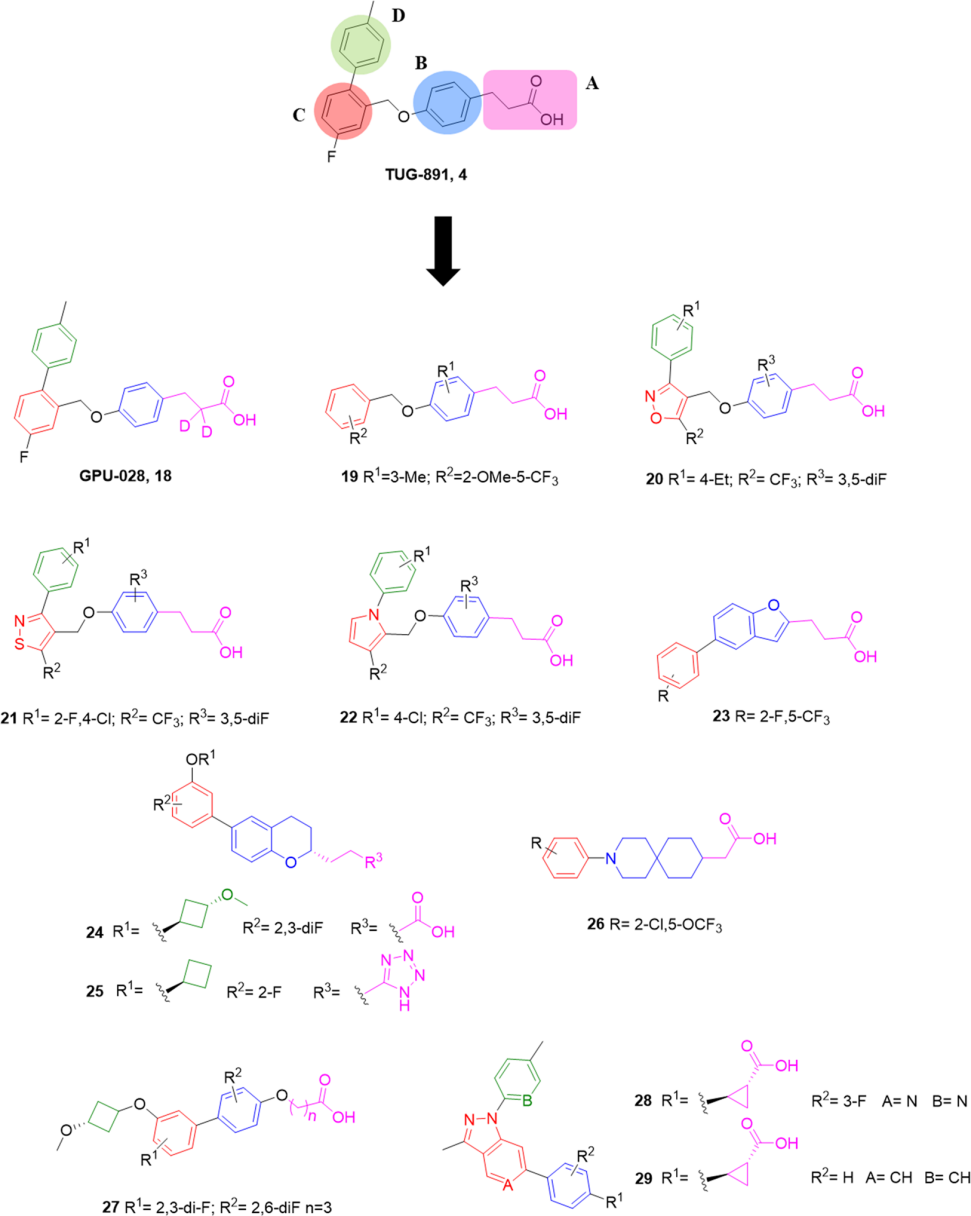
Carboxylic acid-head GPR120 agonists.

The first reported potent and selective GPR120 agonist was compound **4** (Figure 5).^75^ The discovery of **4** was enabled by a refined structural optimization process starting from **3**, a GPR40 agonist with moderate activity against GPR120 (Figure 5).^108^ The structural analogy of several GPR40 agonists with **3** led to the investigation of this general backbone to obtain selective GPR120 activators. First, the effect of some substitutions on the phenyl ring proximal to the carboxylic head of **3** and the replacement of the N-linker with the O-linker were evaluated (Figure 5). Optimization processes that led to compound **3** afforded new derivatives, demonstrating how the presence of a terminal phenyl substituent in the meta position furnishes suitable results in terms of efficacy, but not selectivity, against GPR120.^75^ Conversely, the presence of the same terminal phenyl substituent in the ortho position increased the GPR120 selectivity (Figure 5), as shown by the activity of **4**. Further decoration of the biphenyl moiety with other groups can modulate potency; in fact, typical features of **4** are a fluorine substituent on the phenyl ring proximal to the carboxylic head and a methyl group in position 4 of the *ortho*-terminal phenyl ring, which confer great potency and selectivity versus GPR40, compared to the nonmethylated analogue (Figure 5). Compound **4** achieved an excellent potency and a 1478-fold selectivity for GPR120 over GPR40 (GPR40 pEC_50_ β-arrestin = 4.19), with pEC_50_ of 7.36 (104% efficacy) in a β-arrestin 2 interaction bioluminescence resonance energy transfer (BRET) assay, and pEC_50_ of 7.02 (114% efficacy) in a calcium assay.^75^ As shown in a homology model of GPR120 complexed with **4** (using the crystal structure of the nanobody-stabilized active state of β2-adrenoceptor as a template),^76^ the carboxylic acid moiety interacted with Arg99 through a double hydrogen-bond interaction, with the phenylpropionate with Phe304 and Phe311 in the upper and bottom side, respectively. The biphenyl portion entered in a lipophilic pocket delimited by Met118, Thr119, Gly122, Phe211, Asn215, Ile280, Ile281, and Trp277.^76^ Further studies concerning the potential pharmacological properties of **4** demonstrated that it produced therapeutic effects similar to **1** in murine cell systems; specifically, **4** increased glucose uptake in adipocytes and inhibited pro-inflammatory mediators release.^76^ Moreover, **4** was able to promote a statistically significant GLP-1 secretion from STC-1 cells at 30 μM compared to **1** (100 μM).^109^ Over the years, many **4**-related compounds were developed through modifications or replacements of the phenylpropanoic acid moiety (portions A and B, Figure 6) as well as the biphenyl system (portions C and D, Figure 6). In an additional attempt to improve the metabolic stability (β-oxidation), and thus the pharmacokinetic profile in terms of half-life and clearance, the hydrogens in the α position of the carboxylic acid of **4** (GPU-028, **18**) were replaced with deuterium.^110^ The activity on hGPR120 in the β-arrestin 2 assay is similar for both compounds (EC_50_ of 75.3 nM for **4** and 63.1 nM for **18**).^110^ Additionally, during a four-week study on mice in diet-induced obesity (DIO), the antidiabetic effects of both compounds were analyzed: **18** produced a significant reduction in glucose levels, similarly to **4**, compared to the control.^110^ The aim of many researchers used to be mainly focused on the discovery of new GPR40 ligands; instead, in the past few years, attention has been paid to the implication of GPR120 in T2DM.^30^ In this context, Sparks and colleagues prepared a library of phenylpropanoic acid derivatives to identify new potential GPR120 agonists for the treatment of T2DM.^111^ A structural simplification strategy was used to generate new compounds: the *ortho*-terminal ring present in **4** (portion D, Figure 6) was removed, and the effect of several substituents on the benzyloxy moiety (portion C, Figure 6) and phenylpropanoic acid backbone (portions A and B, Figure 6) was studied. The introduction of a hydroxyl or a methyl group, in position 3 of the propanoic acid chain, abolished the activity in both human GPR120 and GPR40 (calcium mobilization assay).^111^ The presence of two methyl groups in 2,3 or 3,5 positions on the aromatic ring of phenylpropanoic acid portion gave a similar activity compared to the unsubstituted ring (EC_50_ = 304–681 nM), while a single 3-methyl substituent improved the activity along with high selectivity for GPR120 over GPR40. Compounds with monosubstitutions in the para position on the benzyloxy moiety presented moderate activity, while substituents in ortho/meta or disubstitutions in ortho–meta/meta–meta positions were well tolerated (EC_50_ = 40–299 nM).^111^ Among these, compound **19** (2-MeO-5-CF_3_ derivative, Figure 6) presented an EC_50_ value of 299 nM (hGPR120 Ca^2+^ assay) (Table 1) and was selected for *in vivo* antidiabetic studies because of its excellent oral bioavailability and moderate half-life in C57BL/6J mice (*t*_1/2_ = 1.7 h), in combination with good selectivity for human and mouse GPR120/GPR40 (40 and 80-fold respectively, GPR40 Ca^2+^ EC_50_ = 11803 nM) (Table 1). The effect of **19** on the modulation of plasma glucose was examined into two rodent models of T2DM. In Zucker diabetic fatty rats, after 2 weeks of administration of **19** at 10 and 100 mg/kg, there was a reduction of whole blood glucose levels from 192 mg/dL (vehicle control) to 151 and 139 mg/dL, respectively. In db/db mice, **19**, at the same concentrations, decreased glucose levels to 106 mg/dL compared to vehicle control (276 mg/dL).^111^

**Table 1:** Selectivity Data for Phenylpropanoic Acid Derivatives

	hGPR120		hGPR40	
comp.	EC_50_^a^/pEC_50_^b^ (Ca^2+^)	EC_50_^a^/pEC_50_^b^ (β-arr)	EC_50_^a^/pEC_50_^b^ (Ca^2+^)	EC_50_^a^/pEC_50_^b^ (β-arr)
**4**	44/7.02^b^	7.36^b^	65000	4.19^b^
**18**	-c	63.1	75900	-
**19**	299	-	11803	-

a nM.

b pEC_50_ value.

c Data not registered for the referred compound.

#### 5.1.2 Heterocycle Phenylpropanoic Acids

On the basis of the phenylpropanoic acid backbone, recently a set of compounds characterized by different heterocyclic moieties was screened to discover GPR120 agonists. In a study conducted by Zhang and colleagues,^112^ starting from **4**, a new isoxazole-based phenylpropanoic acid series was designed and assayed. Several modifications were introduced to partially reduce the hydrophobicity and optimize the potency versus GPR120. The most relevant difference compared to **4** concerned the replacement of the *meta*-fluoro-phenyl nucleus (portion C, Figure 6) with a five-membered heterocycle, keeping the terminal phenyl ring and the phenylpropanoic acid core in positions 3 and 4, respectively (**20**, Figure 6).^112^ In this context, the presence of several polar diversified heterocycles was evaluated, furnishing new responsive molecules at calcium flux (human GPR120 transfected HEK293 cells) and β-arrestin (PathHunter CHO-K1 cell line expressing human GPR120) assays. In particular, imidazole, triazole, and tetrazole rings suppressed the activity against hGPR120, conceivably because of their excessive polarity; on the other hand, the presence of isoxazole was well tolerated (EC_50_ ranged from 81 to 217 nM in calcium assay) but showed moderate microsomal stability.^112^ Small substituents in position 5 of isoxazole (methyl or ethyl groups) provided an increase in hGPR120 potency compared to unsubstituted analogues, whereas sterically bulky groups decreased the potency. In particular, the presence of a 5-CF_3_ group coupled with a 3,5 di-F substitution on the phenylpropanoic acid moiety and an ethyl group in the para position of the terminal ring afforded the best compound of the series (**20**, Figure 6).^112^**20** showed a GPR120 potency comparable to **4** (EC_50_ = 57 and 60 nM, in Ca^2+^ and β-arrestin assays respectively) (Table 2) with a good pharmacokinetic (PK) profile and *in vivo* hypoglycemic activity in C57BL/6J mice. It was also able to dose-dependently reduce plasma glucose levels with an area under the curve (ΔAUC) of 54% at 10 mg/kg (35% at 3 mg/kg, 30% at 1 mg/kg) in the intraperitoneal glucose tolerance test (IPGTT).^112^ The interesting GPR120 agonism demonstrated by isoxazole phenylpropanoic acid derivatives, together with their moderate microsomal stability,^112^ prompted Zhang and colleagues^113^ to further explore this promising scaffold. The subsequent series proposed by the same authors is constituted by a phenylpropanoic acid moiety linked *via* an ether moiety to an isothiazole nucleus connected to other aromatic rings (**21**, Figure 6). The presence of nonpolar CF_3_ or methyl groups in position 5 of the isothiazole nucleus boosted the hGPR120 potency in both a calcium flux assay (HEK293 cells) and β-arrestin assay (CHO-K1 cells) compared to unsubstituted analogue, while the presence in the same position of polar groups, such as -OCH_3_, decreased the potency.^113^ The replacement of isothiazole with its isomer was also examined, and all compounds showed good EC_50_ values in a calcium mobilization assay (191–94 nM). Furthermore, keeping a 5-CF_3_ substituent on the isothiazole ring and, similarly to **20**, introducing two fluorine atoms in position 3 and 5 of the phenylpropanoic acid portion, together with the double introduction of halogens on the distal phenyl ring, led to an increase of the potency on hGPR120 (EC_50_ = 42 nM in Ca^2+^ assay) with high selectivity over GPR40 (EC_50_ > 5 μM) (**21**, Figure 6).^113^**21** proved to be the most active against human and mouse GPR120 (Table 2) in a calcium flux assay and showed less hGPR40 activity. It reduced in a dose-dependent manner plasma glucose levels in C57BL/6DIO mice subjected to an oral glucose tolerance test (OGTT), with a ΔAUC of 61% and 83% at 1 and 3 mg/kg, respectively, compared to the positive control of saxagliptin (87% at 1 mg/kg). This derivative also presented a good pharmacokinetic profile.^113^ Further investigations on five-membered heterocycles as new scaffolds for the preparation of potential GPR120 agonists were carried out by the same research group.^114^ In this case, the examination was mainly focused on the impact of the pyrrole in place of the phenyl ring C of **4** (Figure 6) and its modifications through the hGPR120 calcium mobilization assay in the HEK-293 cell line. Unsubstituted and 3-halogenated pyrrole derivatives displayed a similar moderate activity (EC_50_ = 161–173 nM), while the presence of a 3-CF_3_ substituent improved the potency on hGPR120 (EC_50_ = 88–91 nM).^114^ Regarding the N-aryl moiety, ethyl and methyl groups in the para position were well tolerated as a chlorine atom (EC_50_ = 50–117 nM). These features in combination with the difluorinated phenylpropanoic acid moiety gave the best active compounds, such as **22** (Figure 6).^114^ It is important to note that the reduction of the carbonyl group to an alcoholic one doubled the potency (EC_50_ = 80 vs 43 nM); anyway, the alcoholic derivative was discarded for PK studies due to its preliminary absorption, distribution, metabolism, excretion (ADME) results. **22** demonstrated a low clearance and suitable half-life in the mouse, rat, and dog.^114^ EC_50_ values in the hGPR120 calcium assay (high-expressing HEK-293 transfected cells and low-expressing endogenous HT-29 cell line) were 80 and 137 nM, respectively (193 nM in mouse), while in the hGPR120 β-arrestin assay (CHO-K1 cell line) **22** showed an EC_50_ value of 69 nM. **22** also exhibited 42- fold (human) and 18-fold (mouse) GPR120 selectivity (over GPR40, in calcium assay, hGPR40 EC_50_ = 3340 nM Ca^2+^ assay) (Table 2). In the OGTT test in diet-induced obese mice, **22** reached a reduction in glucose levels at 3 mg/kg comparable to saxagliptin at 1 mg/kg (positive control). At last, the IPGTT in GPR120 KO and wild-type mice confirmed that these decreased glucose levels were due to the activation of GPR120.^114^

**Table 2:** Selectivity Data for Heterocycle Phenylpropanoic Acid Derivatives

	hGPR120		hGPR40	
comp.	EC_50_^a^ (Ca^2+^)	EC_50_^a^ (β-arr)	EC_50_^a^ (Ca^2+^)	EC_50_^a^ (β-arr)
**20**	57	60	-b	-
**21**	42	143	>5000	-
**22**	80	69	3340	-

a nM.

b Data not registered for the referred compound.

#### 5.1.3 Bicyclic *n*-Carboxylic Acids

As previously reported, the phenylpropanoic acid backbone (Figure 5) showed an interesting behavior as a scaffold for the development of GPR120 agonists.^75,110−114^ Additional studies were conducted on this general chemical structure, modifying the phenyl ring or the chain, with the aim of the development of other suitable agonists. In a study conducted by Merck researchers,^115^ the phenyl ring of the phenylpropanoic acid moiety (portion B, Figure 4) was substituted with a benzofuran core (as in compound **23**, Figure 6). Benzofuran propanoic acid derivatives were prepared starting from an ultrahigh-throughput screen with the objective to identify some lead compounds selective for GPR120.^115^ In this case, compounds with fluorine atoms on the terminal phenyl ring bound to the benzofuran nucleus also showed improved potency in the series, in human and mouse IP1 assays (EC_50_ = 20–63/7–43 nM vs EC_50_ = 474/487 nM). The combination *ortho*-F and *meta*-OCF_3_ afforded the best result (**23**, Figure 6), with IP1 EC_50_ values of 63 (GPR120) and 1829 (GPR40) nM (Table 3). Other modifications of the propanoic acid chain were attempted, but they were generally not tolerated, except for the α-methylation that maintained the activity (EC_50_ = 83 nM).^115^

**Table 3:** Selectivity Data for Bicyclic *n*-Carboxylic Acids

	hGPR120			hGPR40		
comp.	EC_50_^a^ (Ca^2+^)	EC_50_ (β-arr)	EC_50_ (IP1)	EC_50_ (Ca^2+^)	EC_50_ (β-arr)	EC_50_ (IP1)
**23**	-b	-	63	-	-	1828
**24**	-	24	35	-	-	>10000
**25**	-	84	220	-	-	>10000
**26**	-	66	98	-	-	>10000
**27**	93	-	-	>100000	-	-
**28**	740	-	-	>100000	-	-
**29**	360	-	-	>100000	-	-

a nM.

b Data not registered for the referred compound.

**23** displayed good potency against h/mGPR120 with a moderate percentage of receptor activation (73 and 75% respectively) and was 29-fold selective over hGPR40. Furthermore, it proved to have suitable PK properties in the mouse, in terms of oral bioavailability, half-life, and plasma clearance. The OGTT in wild-type and GPR120 KO mice demonstrated an acute reduction of blood glucose levels induced by **23** at both tested doses of 30 and 100 mg/kg.^115^ Starting from the promising results of compound **23**,^115^ the subsequent optimization process consisted of the replacement of the benzofuran moiety with a chromane system, characterizing compounds **24**–**25** (Figure 6).^116^ The effect of several modifications on the chromane propanoic acid chain furnished compounds with abolished activity in both hGPR120 IP1 and β-arrestin assays (CHO-K1). Chromane enantiomers R and S were well tolerated and so was the switch to a cyclopropanoic acid (EC_50_ = 69–160 nM in the IP1 assay), although bioisosteres such as tetrazole reached worse potency. Two series of R-chromane propanoic acid and tetrazole derivatives were then prepared to examine the effect of substituents on the terminal phenyl ring bound to the chromane nucleus.^116^ The presence of one or two fluorine atoms and the meta substitution with a cyclobutoxy group determined an improvement in the potency in both series along with a high selectivity for GPR120 over GPR40. Selected chromane propanoic acid derivative **24** (GPR120 β-arrestin EC_50_ = 24 nM, IP1 EC_50_ = 35 nM) and chromane tetrazole derivative **25** (GPR120 β-arrestin EC_50_ = 84 nM, IP1 EC_50_ = 220 nM) (Figure 6) (Table 3) displayed in OGTT a good *in vivo* efficacy. In particular, **24** dose-dependently reduced glucose levels at 3 and 10 mg/kg, demonstrating a good PK profile in several species, with high oral bioavailability and a long half-life.^116^ The spirocyclic system represented an interesting scaffold to further explore SARs of new GPR120 agonists. Compound **26** (Figure 6) was a selective GPR120 agonist with reported chronic anti-inflammatory properties in obese mice.^117^ It consisted of a spiropiperidine core connected with an ethanoic acid chain and an N-aryl 2,5 disubstituted group (**26**, Figure 6).^117^ Considering its promising results, **26** was selected as a tool for the development of a new set of spirocyclic derivatives.^118^ On the basis of typical features of previously reported agonists, the ortho and meta substitutions on the N-aryl moiety were early preferred; -OCF_3_ in meta positions together with *ortho*-F or -CN substituents afforded moderate potency in human and mouse IP1 and β-arrestin assays (h/mIP1 EC_50_ = 130/49 nM and 200/66 nM, respectively),^118^ while the compounds with nonsubstituted meta positions (EC_50_ > 10000 nM in all studies) were generally inactive; even the replacement of *meta*-OCF_3_ with -OCH_3_ or -CF_3_ (h/mIP1 EC_50_ = 2100/570 nM and 1200/590 nM respectively) was not tolerated. Regarding the acid chain, any changes in length or branching reduced the potency against GPR120. **26** afforded the highest potency on GPR120 in both hIP1 (EC_50_ = 98 nM) and hβ-arrestin (EC_50_ = 66 nM) and poor activity against hGPR40 (at last 102-fold selective for hGPR120, EC_50_ > 10 000) (Table 3).^118^ Therefore, the OGTT in lean mice (wild-type/GPR120 KO) was performed, and compound **26** dose-dependently reduced whole blood glucose levels at 30 and 100 mg/kg. Further evaluation of the insulin sensitivity improvement, in a hyperinsulinemic-euglycemic DIO mouse clamp, demonstrated that **26** produced an increase in insulin levels together with a reduction in insulin resistance (HOMA-IR) on days 14 and 28. Unfortunately, PK studies in rat, mouse, dog, and Rhesus displayed a too high unbound clearance.^118^

Sheng and colleagues described a series of biphenyl butanoic acid derivatives as new selective GPR120 agonists.^119^ The presence of two phenyl rings as a bicyclic system, connected by an O-linker to the butanoic acid chain (**27**, Figure 6), was useful to obtain good results in an hGPR120 calcium influx assay (CHO cells). The introduction of mono- and disubstitutions on both phenyl rings allowed the identification of critical features for improved potency.^119^ In fact, concerning the terminal phenyl ring, the NO_2_ group in the ortho position dramatically reduced the activity on both hGPR120 and hGPR40, while the presence of the methylenedioxy group (position 2–3) confirmed a moderate activity on GPR120 (EC_50_ = 200 nM). The presence of the methoxy substituent on the proximal phenyl ring completely abolished the agonist activity.^119^ The simultaneous presence of two fluorine atoms *per* ring, together with a cyclobutyloxy substituent in the terminal one, afforded the most promising results (**27**, Figure 6) in terms of activity and selectivity over GPR40, similarly to other active fluorinated compounds (GPR120 Ca^2+^ EC_50_ = 93 nM, GPR40 Ca^2+^ EC_50_ > 100 000 nM) (Table 3). Furthermore, modifications of the acid chain highlighted the relevance of the chain length. Too short chains were not well tolerated, while a C7–C8 chain length resulted in an increased potency against hGPR40 (GPR120 EC_50_ = 24.04 μM, GPR40 EC_50_ = 3.48 μM). Noteworthy, **27** showed its hypoglycemic properties (OGTT test) in ICR mice, displaying a dose-dependent reduction of glucose levels at 10 mg/kg.^119^ Molecular modeling studies were performed^120^ to explore the protein–ligand interactions between **27** and GPR120/GPR40. GPR120 in complex with **27** showed a typical and already reported hydrogen-bond interaction between Arg99 and the oxygen of carbonyl group, but it proved to be not stable due to the distance between nitrogen and carboxylic function; in fact, this interaction disappeared in the equilibrated state (MD simulation). The other two identified residues which hid Arg99 and established hydrogen-bond interactions with **27** were Trp104 and Trp299. Arg99 seems to have a key role in the stabilization of these residues in their positions.^120^ To increase the structural rigidity of typical small molecule GPR120 agonists, a more complex set of biphenyl derivatives was developed by McCoull and colleagues,^121^ which was chemically characterized by a condensed-pyrazole core bearing a 6-phenyl substituent and a N-aryl/heteroaryl moiety (**28**–**29**, Figure 6). The ethanoic acid chain proved to be inactive, while 3- and 4-carbon chains with an unsubstituted N-aryl moiety were tolerated (EC_50_ = 0.64–0.26 μM) but showed a low selectivity (hGPR120 calcium flux in CHO cell line).^121^ For these reasons, a cyclopropyl carboxylic acid function was inserted, and the stereochemistry effect was analyzed, highlighting the activity of only the *S,S*-enantiomer (EC_50_ = 0.69 μM vs >17 μM). One of the best two selective compounds was generated from the combination of two pyridine rings and a 3-F substituent on the phenyl at position 6 of the condensed-pyrazole bicyclic nucleus (**28**, Figure 6).^121^ The other one is made up of two simple phenyl rings, compared to pyridine ones, with no halogens at the 6-phenyl substituent of the condensed-pyrazole core (**29**, Figure 6, Table 1). **28** and **29** displayed selective hGPR120 activity (GPR120 Ca^2+^ EC_50_ = 740 and 360 nM, respectively) and high selectivity over muGPR40 (EC_50_ > 100 000 for both compounds, 135-fold, in FLIPR format for overexpressed mouse GPR40 in HEK923s cell line), probably due to the noticeable rigidity of the structure (Table 3).^121^ Both **28** and **29** showed a decrease in oral glucose excursion (45% and 65% respectively) in OGTT (C57BL/6J mice); in wild-type mice, they promoted a similar reduction (47% and 58%, respectively), while no effect was observed in GPR120 KO mice. In addition, both compounds presented moderate oral exposure and good selectivity over 30 several targets.^121^

### 5.2 Nonacidic Head Derived Agonists

The data reported until now evaluated the typical features of GPR120 agonists, represented by a carboxylic head, an alkyl-heteroaryl chain, and a diversified aromatic tail (Figure 6).^67,103−114^ In this context, the search for new GPR120 ligands prompted various scientists to evaluate plausible variants of the carboxylic head (such as the hydroxyl group) or isosteric substituents such as the sulfonamide one.

#### 5.2.1 Phenyl-Propyl Alcohols

**Figure 7:**
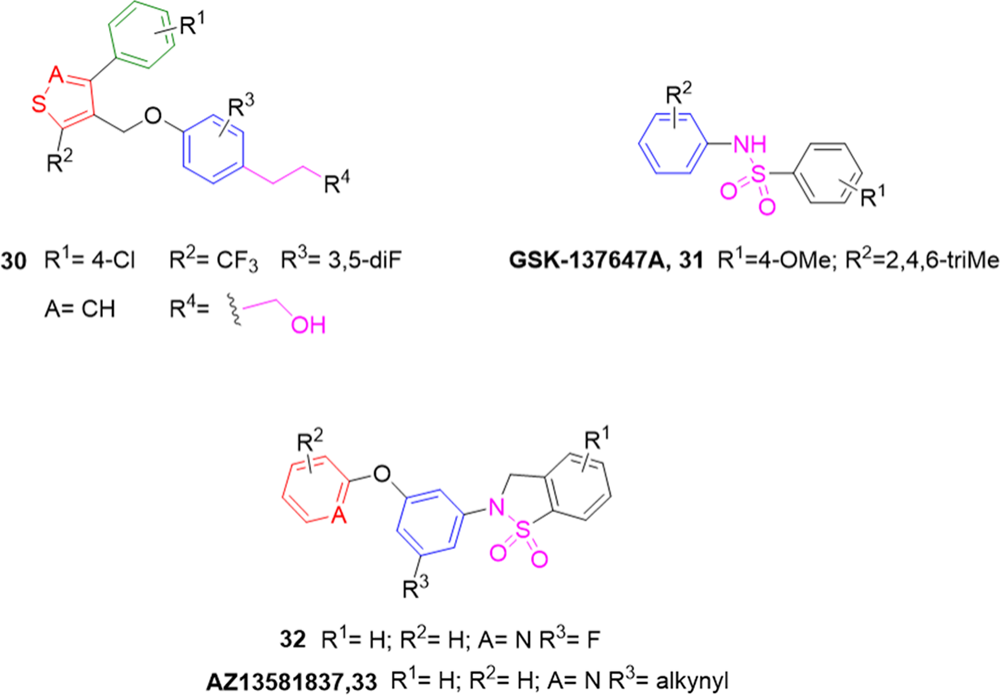
Nonacidic headgroup GPR120 representative agonists.

According to literature data, in a recent patent (US9045454B2),^122^ variegated isothiazole and thiophene derivatives have been demonstrated to be GPR120 agonists, prepared with the aim of being useful tools for the treatment of different GPR120-mediated disorders. The typical structure (**30**, Figure 7) includes the main modification suggested by providing the best GPR120 affinity. Briefly, in this structure two heterocycles, such as thiophene (A = C) and isothiazole (A = N), were selected as central core, linked through an ether bridge to a phenyl-propyl, acidic, or alcoholic chain.^122^ Several substituents in the 5-position of the heterocycle have been evaluated (cyclopropyl, 1,1-difluoroethyl, trifluoromethyl, and phenyl groups) and showed how the trifluoromethyl group is present in the most active molecules. Among the compounds suitable for *in vivo* studies, the head-alcoholic compound **30** demonstrated hGPR120 EC_50_ values of 125 nM (β-arrestin) and 165 nM (Ca^2+^) in human GPR120 discoveRx PathHunter beta-arrestin and *in vitro* human GPR120 calcium flux assay, respectively (Table 4). In GPR120 DIO mice OGTT and GPR120 C57BL6 mice IPGTT **30** showed positive outcomes from both assays confirming the therapeutic validity of this series of synthetic compounds.^122^

**Table 4:** Selectivity Data for Nonacidic Head Derived Compounds

	hGPR120		hGPR40	
comp.	EC_50_^a^/pEC_50_^d^ (Ca^2+^)	EC_50_/pEC_50_^d^ (β-arr)	EC_50_/pEC_50_^d^ (Ca^2+^)	EC_50_/pEC_50_^d^ (β-arr)
**30**	165	125	-c	-
**31**	6.3^d^	-	<4.5^d^	-
**32**	6.63^d^	6.91^d^	NA^b^	-
**33**	120	5.2	-	-

a nM.

b NA: not active at 100 μM.

c Data not registered for the referred compound.

d pEC_50_ value.

#### 5.2.2 Sulfonamide Derivatives

From a medicinal and chemical point of view, a typical replacement of the carboxylic acid moiety could be a sulfonamide residue, investigated even in some studies for the development of GPR120 ligands. This seemed to be also in line with other FFARs ligands that presented a sulfonamide functionality, able to interact with Arg residues in the active sites.^19^ GSK researchers prepared diarylsulfonamide derivatives,^123^ with a general structure devoid of the typical carboxylic acid moiety, in which the sulfonamide function is connected with two phenyl rings variously substituted (**31**, Figure 7). The effects of the substituents on both aryl groups were examined using a 10-point response curve in the U2OS cell line expressing hGPR120. Regarding the aryl-sulfonyl group, para-substitutions were generally preferred compared to ortho-substitutions (no activity).^123^ In particular, nonpolar groups in the para position were tolerated, and the presence of 4-OMe led to suitable potency and 100% max response; conversely, 4-Me and 4-OCF_3_ displayed a decrease in efficacy (83% and 53% max response, respectively). The insertion of monoalkyl-substituents on the aniline core suppressed the activity, while disubstitutions at the 2,4 and 2,6 positions showed moderate activity and efficacy (hGPR120 pEC_50_ = 5.6–81% and 5.5–63%, respectively). The trimethyl substitution with the simultaneous presence of the 4-OMe-aryl-sulfonyl ring afforded the most promising compound, **31**, known as GSK137647A (hGPR120 Ca^2+^ pEC_50_ = 6.3, Figure 7) (Table 4).^123^ The selectivity of **31** for GPR120 over more than 65 targets, including GPR40, GPR43, GPR41, was evaluated and resulted from at least 100-fold selectivity. Unfortunately, **31** proved to have weak solubility in simulated intestinal fluid (FASSIF), limiting its druggability.^123^ In glucose-stimulated insulin secretion (GSIS, glucose concentration 25 mM) in the MIN6 cell line, **31** dose-dependently increased insulin output compared to positive control glibenclamide. **31** also evoked GLP-1 secretion in the human intestinal NCI-H716 cell line. Despite these promising results, further modifications should be introduced into the GSK-developed structure to improve its low solubility as well as to find new potent GPR120 nonacidic agonists.^123^ In this context, a set of cyclic and less polar sulfonamides were later prepared and evaluated for GPR120 agonism in β-arrestin 2 (HEK 293 cells) and calcium mobilization (Flp-In T-REx 293 cell lines) assays.^124^ The general structure included a benzosultam core with an N-aryl moiety bearing a terminal aromatic/heteroaromatic ring (**32**, Figure 7). Several modifications were introduced to examine the impact of sulfonamide functionality; consequently, removing the sulfonamide reduced the activity, while cyclic sulfonamide derivatives showed better activity in general than acyclic ones.^124^ On the other hand, the terminal phenyl ring with nonpolar groups, such as Me or CN, conferred moderate activity on both hGPR120 (β-arr. pEC_50_ range = 5.57–6.36 and 5.24–5.78, respectively) and hGPR40 (Ca^2+^ pEC_50_ range = 4.35–5.54 and 4.70–6.21, respectively) but less than when the aromatic ring was a pyridine (**32**, Figure 7, Table 4). **32** showed an EC_50_ value of 198 nM, with high selectivity for GPR120 over GPR40 (>300-fold selective) but relatively low solubility in PBS.^124^ The antidiabetic activity of **32** in OGTT (C57BL6 mice) was evaluated, and it produced a decrease in glucose levels (orally prior administration) at 10 mg/kg compared to vehicle control. In a chronic study in DIO wild-type and GPR120 KO mice, **32** reduced glucose levels and increased the insulin sensitization in wild-type mice (murine insulin ELISA kit), complemented by a decrease in body weight (7–9%); no effect was observed in mice lacking GPR120.^124^ A close structural analogue of **32** was synthesized^125^ as a potent GPR120 agonist, in which an alkynyl group was present on the N-aryl moiety (AZ13581837, **33**, Figure 7). In the Ca^2+^ mobilization (CHO-hGPR120 cell line) and β-arrestin (U2OS-hGPR120) assays, **33** displayed EC_50_ values of 120 nM and 5.2 nM respectively, accompanied by the increase of cyclic adenosine monophosphate (cAMP) levels with an EC_50_ value of 60 nM (cAMP assay in a CHO cell line).^125^ It also promoted a dynamic mass redistribution response (in mouse/human GPR120, hGPR120 EC_50_ = 5.2 nM), while no activity was found against mGPR40 (Table 4). Further *in vivo* studies demonstrated that **33** was able to boost GLP-1 secretion in an enteroendocrine STC-1 cell line and reduce dose-dependently glucose levels in lean male mice (OGTT). Finally, in the intravenous glucose tolerance test (IVGTT) in lean mice, pretreatment with **33** evoked an increase of insulin concentration.^125^

### 5.3 Patent Highlights

**Figure 8:**
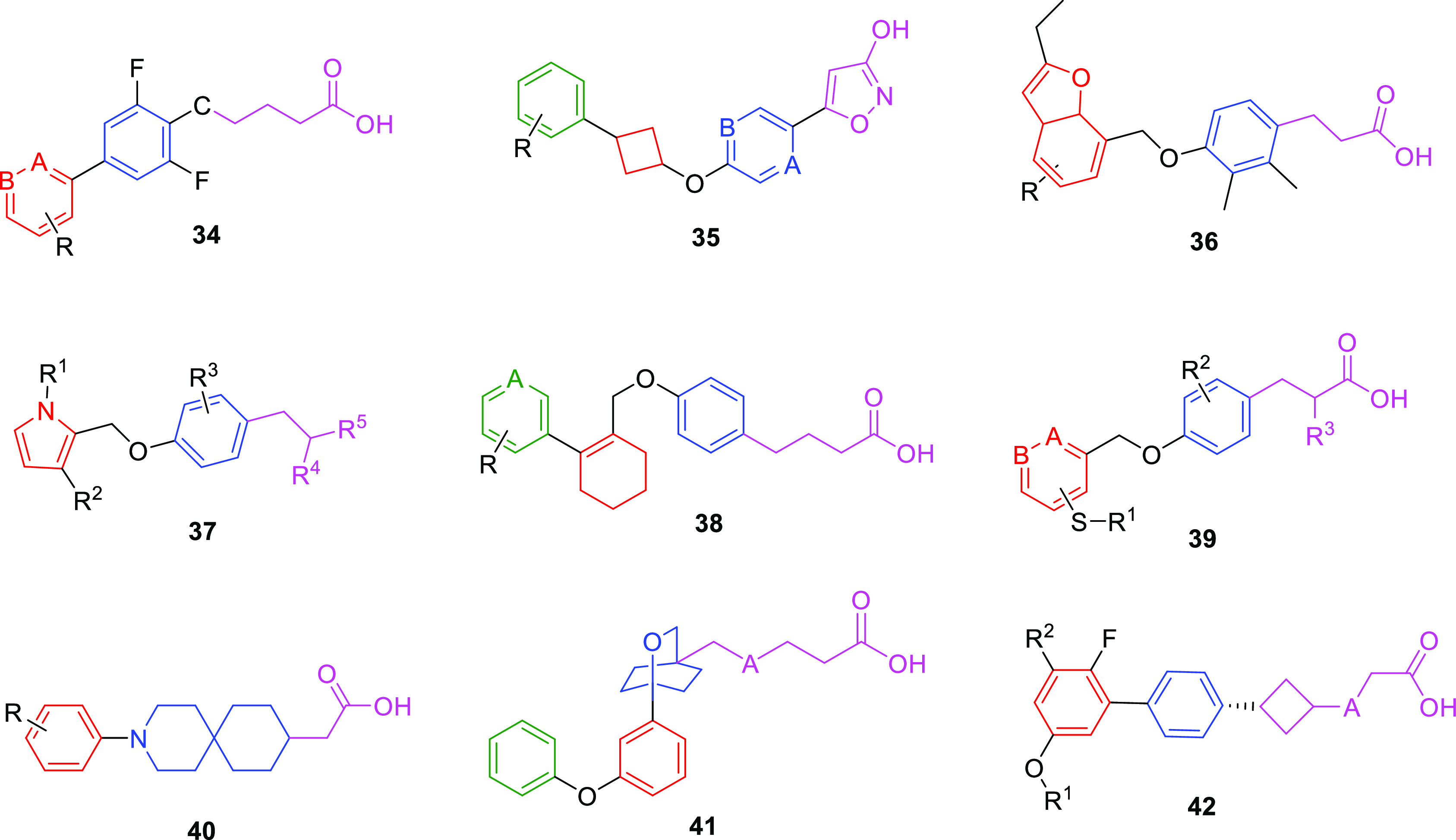
Patented general chemical structure useful GPR120 agonists.

On the basis of pharmacological data, the role of GPR120 agonists in the management of T2DM and other diseases has been proven, including cancer and inflammatory conditions; pharmaceutical companies used this information to find new and selective GPR120 agonists, although in most cases their therapeutic efficacy was not evaluated. LG CHEM, Ltd proposed a library of biphenyl derivatives (US10221138B2) with a general structure, such as **34** (Figure 8). The biphenyl/phenyl-pyridine (A or B = N) backbone represents the core of these structures, frequently bearing two fluorine atoms in the meta-position in one ring and a terminal carboxylic head. They demonstrated interesting values as GPR120 agonists (cell-based assay), and most of them have an agonistic effect at EC_50_ < 0.2 μM.^126^ Substituted isoxazole derivatives have been designed by Merck Sharp & Dohme Corp. (US0269679A1) and assayed as GPR120 modulators. The common pharmacophore motif is represented by an isoxazole nucleus, substituted in C3 and C5 positions. In C3, it is frequently retrieved as a phenol or phenate group, while the C5 position represented the anchoring point of aryl groups, which prolong the spacer throughout ether bridges. The activity against GPR120 was recorded using the h/rGPR120 IP1 assay. Many of the patented compounds (general structure **35**) showed EC_50_ < 10 nM in both assays (Figure 8).^127^ A similar screening was carried out by Janssen Pharmaceutica NV on a wide set of benzo-fused heterocyclic derivatives (US10155737B2) (**36**, Figure 8) that showed promising agonist GPR120 activity.^128^ The fused-cyclic moiety was also retrieved in a recent patent reporting the preparation of a very large set of chemically diverse heterocyclic-fused derivatives (US10214521B2), showing EC_50_ values in an *in vitro* BRET assay that varied between 10 nM to 10 μM.^129^ Parallel to the previous chemical structures, Janssen Pharmaceutica NV proposed a series of patented bicyclic pyrrole derivatives (US9045454B2) of general formula **37** (Figure 8). They are characterized by a phenyl or pyridinyl group as an N-aryl moiety R^1^, wherein the rings are optionally substituted with one to three substituents independently selected from the set consisting of halogen, cyano, carboxyl, or alkyl groups. Substituents in position 3 of the pyrrole ring (R^2^) can be hydrogen, halogen, cyano, C-alkyl, or fluoro-substituted C-alkyl; R^3^ is independently selected from the group consisting of halogen, C_1–4_ alkyl, and fluoro-substituted C_1–4_ alkyl. The terminal alkyl chain could be a hydrogen or a ramification (R^4^) consisting of a methyl group or a polar head (CH–OH or −COOH).^130^ Nevertheless, different structures have been developed and assayed as suitable GPR120 agonists. For example, Piramal Enterprises Limited proposed a library of substituted phenyl alkanoic acid compounds (US10273230B2) of general formula **38** (Figure 8); these derivatives presented a variegated chemical diversity, but several motifs, like an ether bridge and a carboxylic chain, seem to be essential. The phenyl and heterocyclic nuclei are spaced thanks to an ether chain. The butyl chain could be decorated with different ramifications or cyclopropyl moiety, while the pyridine nucleus (A = N) can be usefully substituted in different positions with thiocyclopentyl/hexyl groups. These compounds have been tested for their activity against GPR120 using the β-arrestin 2 interaction assay (BRET assay) performed in CHO-K1 cells using the β-galactosidase (Beta gal) enzyme fragment complementation assay^131^ and showed the best EC_50_ values ranging from 50 nM to 500 nM.

Another class of free acidic head derivatives (general formula **39**, Figure 8) was proposed by LG Life Sciences LTD (WO069963A1), and it is constituted by the typical carboxylic acid chain embedded to a central phenyl ring, which ended up attached through an ether connection to a pyridine ring (A or B = N), bearing different thioether connections. Their GPR120 activity was measured in a CHO-K1 cell line, revealing that most of the new compounds act as agonists with EC_50_ < 0.2 μM.^132^ A wide set of spiropiperidinyl derivatives (general formula **40**) was synthesized as potent GPR120 agonists by Merck Sharp and Dohme Corp (WO059232A2). The usefulness of these compounds was ascertained by the *in vitro* FLIPR assay. The most potent compounds showed an hGPR120 EC_50_ in a range of 300–500 nM. According to SAR, the appended phenyl ring was advantageously decorated in position 3 with methyl or ether groups, mainly trifluoromethoxy. A second substitution could be posed in 4 or 5 positions and should be a chlorine, nitrile, or trifluoromethoxy group. The lateral chain is usually a propionic residue with a free carboxylic head, or a 2-hydroxyethyl moiety (**40**, Figure 8).^133^ Bristol-Myers Squibb Company patented different structures as GPR120 modulators, which may be used as medicaments alone or in combination with other antidiabetic drugs. The AU2014235172B2 patent is related to novel substituted bicyclic acid compounds (**41**, Figure 8), which can modulate GPR120, as measured with pERK activity. The common structural motif is a bicycle endowed with a heteroatom (O), also found in different positions and a free acid chain on the quaternary carbon atom, together with a double bond, or an ether group. A diphenyl ether in all the best active compounds represents the appended aromatic moiety. Compounds with desirable pharmacokinetic properties were evaluated in mice for glucose lowering by monitoring disposition of an oral glucose load by an OGTT test.^134^ As reported in the US10023519B2 patent, the compounds presented nanomolar activities as GPR120 agonists. In particular, the best active compounds are two isomers, i.e., 2-(*trans*/*cis*-3-(2′-fluoro-5′-isopropoxy-[1,1′-biphenyl]4-yl)cyclobutyl) acetic acids. The structures (**42**, Figure 8) displayed the same skeleton with a cyclobutyl ring linked to an aliphatic acid tail and a biphenyl moiety, where one of the two rings is pervaded by halogens (fluorine in the best one) and an aliphatic or aromatic ether group.^135^ US10336684B2 provided novel phenylcycloalkyl and phenylazacycloalkyl carboxylic acid compounds, and their respective analogues. The best activity was observed when a pyrrolidine core is linked to the phenyl pentanoic acid chain, compared to the presence of a six-membered cycle.^136^

## 6 Perspectives

**Figure 9:**
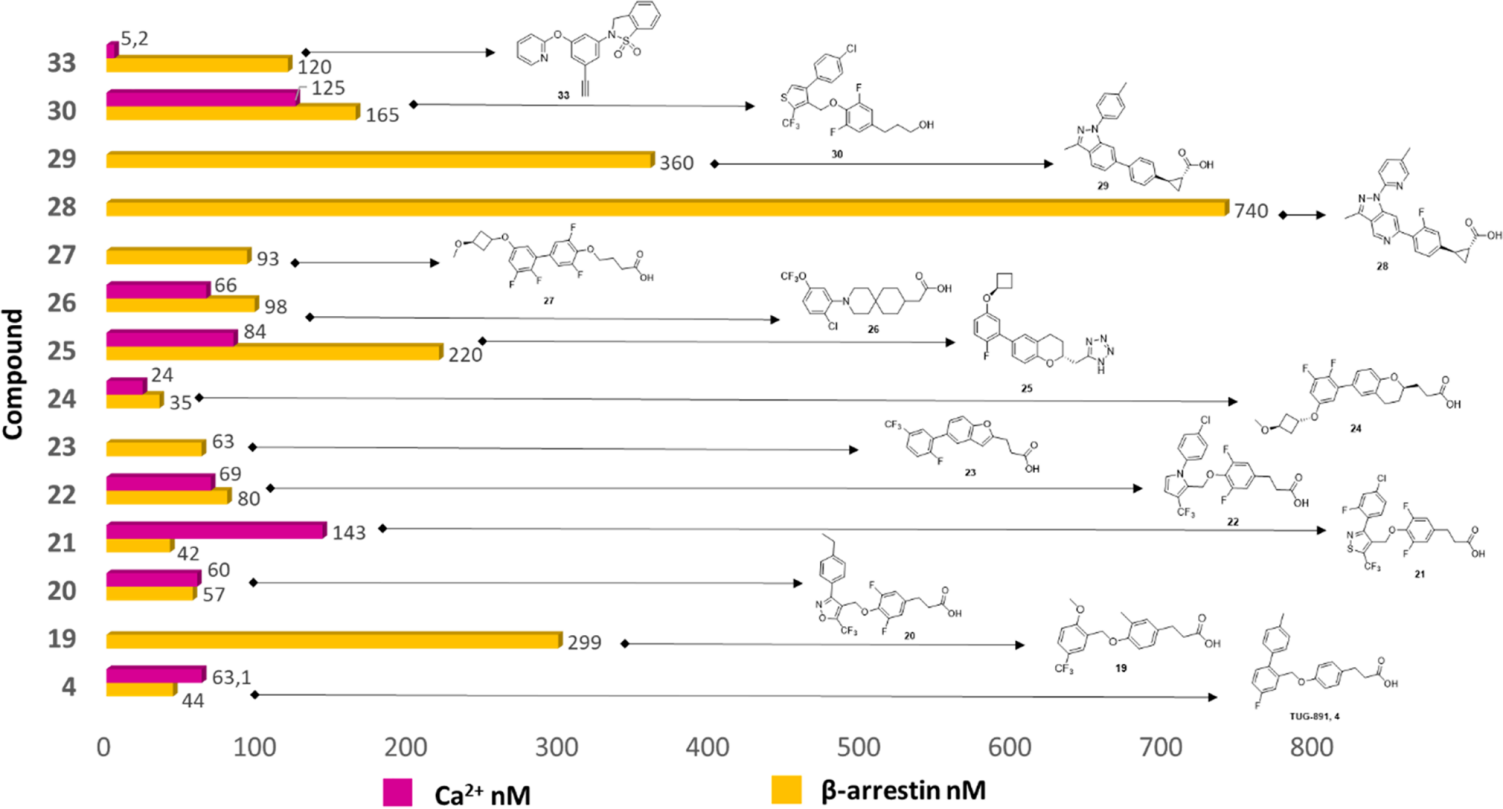
SAR opportunities for GPR120 ligands.

The GPR120 receptor comprises a complex pharmacological activity, with different effects in metabolic disorders. The best studied disease in the context of GPR120 medicinal chemistry is T2DM. Nevertheless, no GPR120 ligands have been approved as antidiabetic drugs.^30^ Nowadays, T2DM is constantly monitored by controlling the diet or by using drugs, until insulin treatment. The main drugs used were able to (i) enhance insulin secretion, (ii) sensitize the target organs of insulin, and (iii) impair glucose absorption.^137^ Metformin (a biguanide) is used as the first line of treatment because it presents a low risk of hypoglycemia and weight gain and is low cost. However, it presents gastrointestinal side effects such as nausea, vomiting, and diarrhea.^138^ Sulfonylureas increase hypoglycemia risk and weight gain. DPP-IV inhibitors improve glycemic control, limiting the risk of hypoglycemia or weight gain. Nonetheless, these drugs increase the incidence of acute pancreatitis in susceptible patients and hospitalization for heart failure.^139^ These data prompted evaluation of new targets for T2DM treatment. In this field, studies have shown how FFARs are drug targets, in particular, the members GPR40 and GPR120. The last one emerged as an intriguing modulator of several physiological functions that highlighted its use as pharmacological template in medicinal chemistry for the development of new drugs. Its high expression in enteroendocrine cells favored its translation from tissue to pharmacological activity, while its activation promoted GLP-1 secretion, which in turn indicated an insulin secretagogue activity in the pancreas, validating its role in T2DM. At first, TUG-891 served as a precursor for many newly carboxylic acid-head-based synthesized compounds. Later, SAR studies revealed also the essential moieties necessary to obtain a good GPR120 agonist, independently from the variously substituted chain. A carboxylic head (responsible for the receptor activation after hydrogen-bond interaction with the guanidine group of Arg99), an aryl/heteroaryl linker, and a diversely decorated tail constitute the typical features of a GPR120 agonist. As depicted in Figure 9, the phenylpropanoic acid moiety linked to heterocycles (as in compounds **20**, **21**, and **22**) maintained the agonistic activity versus GPR120, in a similar manner to compound **4**. When the acid chain was connected with bicyclic systems, as benzofuran in compound **23** or as a spiropiperinidyl moiety in compound **26**, GPR120 agonism was rescued. Moreover, compound **24**, presenting a chromane core, provided a slight optimization in terms of EC_50_, compared to **4**, in both Ca^2+^ and β-arrestin assays. When the chromane system was attached to the tetrazole one, such as in **25**, the activity was reduced mainly in the β-arrestin assay. Conversely, when the terminal phenyl ring of chromane derivative **24** was decorated in the meta position with a cyclobutyloxy moiety, also present in compound **27**, the activity increased considerably. The rigidity shown by compounds **28** and **29** dramatically reduced the GPR120 agonist activity (in the β-arrestin assay), although between the two of them, **29** presented better activity. Interestingly, noncarboxylic acid-head-derived compounds revealed a useful building block for the design of new selective agonists (**30**, **33**). In particular, sulfonamide **33** displayed an excellent activity in a Ca^2+^ assay, revealing how the presence of some groups (generally bioisosteres of carboxylic one) furnished an interesting SAR opportunity. Overall, in the most active compounds, the presence of trifluoromethyl or trifluoromethoxy groups furnished a good efficacy in terms of selectivity despite GPR40. Moreover, considering that fluorination adjacent to atoms with π-bonds increases lipophilicity, this substitution pattern could be an interesting feature in PK. Noteworthy, from the medicinal chemistry point of view, various bioisosteres of carboxylic acid could be used in drug design, such as amides, thiazolidinediones, trifluoromethyl ketones, hydroxamates, and other ones, by varying their size, geometry, charge distribution, acidity, and lipophilicity.

However, the optimal pharmacological outcome should be dependent on the physicochemical and pharmacodynamic properties.^140^ The reported studies highlighted the response to Ca^2+^/β-arrestin and confirmed in most cases the *in vivo* activity in different glucose tolerance tests. Nevertheless, the real big challenge is still open, whether or not biased GPR120 agonists favor one signaling cascade over the other and whether such pathway selectivity may be relevant, which will surely lead to an arduous journey to obtain clinically validated GPR120 agonists. Furthermore, the exact pharmacological route involved in T2DM helps to investigate how GPR120 ligands might be useful tools for other pathologies, including T2DM comorbidities, cancer, and inflammation.

## Abbreviations Used

aLA: α-linolenic acid
AMPK: 5′ AMP-activated protein kinase
Arg: arginine
Ala: alanine
AUC: area under the curve
Bcl-2/Bax: B-cell lymphoma 2/Bcl-2-associated X protein
BRET: bioluminescence resonance energy transfer
CNV: choroidal neovascularization
COX-2: cyclooxygenase 2
DHA: docosahexaenoic acid
DIO: diet-induced obesity
EGF: epidermal growth factor
EGFP: enhanced green fluorescent protein
EPA: eicosapentaenoic acid
ERK1/2: extracellular-signal-regulated kinase 1/2
FFAs: free fatty acids
GIP: gastric inhibitory peptide
GLP-1: glucagon-like peptide-1
GPCRs: G-protein coupled receptors
GSIS: glucose-stimulated insulin secretion
HFD: high-fat diet-fed
ILs: interleukins
IPGTT: intraperitoneal glucose tolerance test
IVGTT: intravenous glucose tolerance test
KO: knockout
LCFAs: long chain fatty acids
LPA: lysophosphatidic acid
LPS: lipopolysaccharide
MAPK: mitogen-activated protein kinase
MCFAs: medium chain fatty acids
NF-κB: nuclear factor kappa B
OGTT: oral glucose tolerance test
PK: pharmacokinetics
PKB/Akt: protein kinase B
PPARγ: peroxisome proliferator-activated receptor-gamma
PUFAs: polyunsaturated fatty acids
SAH: subarachnoid hemorrhage
SAR: structure–activity relationship
SCFAs: short chain fatty acids
T2DM: Type 2 diabetes mellitus
TMDs: transmembrane helical domains
TNF-α: tumor necrosis factor alpha
